# Oil-in-Water Emulsions Made of Pistachio Oil: Physical and Chemical Properties and Stability

**DOI:** 10.3390/foods14010060

**Published:** 2024-12-28

**Authors:** Lama Ismaiel, Valeria Rizzo, Carla Di Mattia, Benedetta Fanesi, Paolo Lucci, Giulia D’Alessio, Deborah Pacetti, Paola Pittia

**Affiliations:** 1 Department of Agricultural, Food and Environmental Sciences, Università Politecnica Delle Marche, 60131 Ancona, Italy; l.ismaiel@univpm.it (L.I.); b.fanesi@pm.univpm.it (B.F.); p.lucci@univpm.it (P.L.); d.pacetti@univpm.it (D.P.); 2 Department of Biosciences and Technology for Food, Agriculture and Environment, University of Teramo, Via R. Balzarini 1, 64100 Teramo, Italy; cdimattia@unite.it (C.D.M.); gdalessio@unite.it (G.D.)

**Keywords:** pistachio oil, technological functionality, emulsion ability, bioactive compounds, emulsion lipid oxidation

## Abstract

Pistachio nuts are valued for their sensory qualities, nutritional benefits, and health-promoting properties. Pistachio oil has also gained interest for its bioactive compounds, though these are sensitive to processing and environmental stresses. While pistachio-based products are commercially available, little research has addressed the emulsifying properties of crude pistachio oil or its impact on the stability and bioactive profile of oil-in-water (O/W) emulsions. This study evaluated the emulsion-forming abilities of two commercial pistachio oils (PO1, PO2), their physical and oxidative stability during emulsification, and the effects of emulsifier concentration over short-term storage (7 days, 4 °C). O/W emulsions were prepared using 20% (*w*/*w*) oil and Tween 20 (0.5% and 1% *w*/*w*) in phosphate buffer and homogenized under high pressure. The emulsions were analyzed for particle size, fatty acid profile, antioxidants, and oxidative state. The results revealed differences in fatty acid composition, oxidative stability, and bioactive content between the oils and their emulsions. PO1 showed higher levels of bioactives compared to PO2. Emulsification increased the peroxide value of the oil phase, confirming its pro-oxidant effects. The results of this study demonstrate the potential of pistachio oil to create stable O/W emulsions rich in bioactives, offering new opportunities for healthy emulsified food products.

## 1. Introduction

Edible tree nuts are valued globally for their sensory, nutritional, and health attributes and economic importance. Pistachio (*Pistacia vera* L.), the only species in its genus with commercial value, is native to West Asian countries and was brought by traders to Syria, Italy, and the rest of the Middle East and Mediterranean countries as well as Europe in the first centuries AD [1,2]. The cultivation of this crop was introduced to the United States in the 19th century. Over the decades, significant growth in production has led California to become the world’s leading producer today. In many countries, a variety of cultivars are used for pistachio production. In the United States and Australia, the female cultivar “Kerman” and the male cultivar “Peter” are the most commonly used. Some cultivars are characterized by peculiar chemical and sensory properties and the European Commission has officially registered the pistachios produced in Sicily in the Bronte area (Italy) as a Protected Designation of Origin product (“Pistacchio Verde di Bronte”) by recognizing its unique quality and reputation [3,4].

The geographical origin of pistachio kernels (*referred as ‘pistachio’ from here onwards in the text*) is identified as a source of variability in the product properties and chemical composition [5,6,7]. Pistachio composition includes its main nutrients, and in particular proteins (ca. 20%), carbohydrates and fibers, minerals, inositol, phosphate, and vitamins. However, the macronutrient quantitatively more present in pistachio nuts is represented by the lipidic fraction, corresponding to 50–62% of its dry weight, with some cultivars presenting content as high as 75% [7,8] with valuable unsaturated fatty acids such as oleic, linoleic, and linolenic ones.

Pistachio is also rich in bioactive secondary metabolites and phytochemicals including phenolic compounds (e.g., gallic acid, catechins, and various flavonoids), chlorophylls, tocopherols, and carotenoids, exhibiting significant antioxidant and radical-scavenging abilities [1,9,10]. Studies have highlighted that the pistachio skin of the kernel contains significantly higher total phenolic content compared to virgin pistachio oil extracted from the seed [11]. However, the low polarity and hydrophilicity of the major phenolics, along with that of some other antioxidants of the seeds, may hinder their partition in the oil, that, thus, in general, could be characterized by a lower bioactive potential [12]. The phenolic content of pistachio nuts may depend on cultivar, geographical origin, ripening stage, and industrial processing, ranging from 73 mg kg^−1^ to 138 mg kg^−1^ [13] and 16 to 23 mg kg^−1^ [14]. Sena-Moreno et al. [15] reported that the total phenolic content is also influenced by the drying temperatures applied to the pistachios prior to the oil extraction, and values in the range from 38 to 60 mg kg^−1^ GAE in samples dried around room temperature were found.

Among tocopherols, γ-tocopherol represents the main isomer in pistachio oils [1,2], while lutein was found to be the main carotenoid. Total carotenoid content varies significantly from ca. 1.5 to 4.7 mg 100 g^−1^ product [13,16,17,18] with the higher values determined in products from the U.S. and Sicily and, in particular, in Bronte pistachios, while lower values were found in the pistachio oil of East Azerbaijan (5.4–11.5 mg kg^−1^ oil) [7]. Pistachio oil also presents a notably higher phytosterol content (ranging from 2100 to 7600 mg kg^−1^ oil) compared to virgin olive oil (1100 to 2100 mg kg^−1^ oil) [12].

The high health potential of pistachio oil related to the richness of phytochemicals could be, however, easily impaired by the processing actions applied to nuts and kernels due to their sensitivity to thermal and other technological stresses. Drying, oil extraction, and other processing actions have already been recognized as causing a decrease in both content and biological activity of healthy compounds [15,17].

Pistachio oil, that could also be extracted from pistachios not fitting the quality standards for fresh consumption [2], represents an interesting lipidic source, thanks to its distinctive nutritional traits and sensory qualities, to be used both as an added value oil compared to other vegetables ones [14] and as an ingredient for innovative and healthy formulated emulsified food products. Different pressed nuts and their products have been reported as high value-added compounds in food fortification [19].

In food production, emulsification is a main technological action that permits the incorporation and structuring of immiscible components (e.g., oil, lipids, and hydrophobic compounds) in a formulated food thanks to the contribution of surface-active compounds (emulsifiers) that play a main role in the improvement of physical, nutritional, and sensorial properties of the final products. In food emulsions, the attainment and maintenance of an adequate structure and the achievement of oxidative and physical stability upon storage represent a key goal due to the intrinsic thermodynamic and kinetic instability [20]. Many food products are made of oil-in-water (O/W) emulsions, and their properties greatly influence the quality of food products [21,22]. The physical and structural properties of an O/W emulsion depend on several factors, including the nature and concentration of the dispersed oil, the volume and distribution of the oil droplets, their interactions with other ingredients, the type and concentration of the emulsifier and the method of homogenization. Changes in any of these factors can alter the emulsion’s rheological, textural, and sensory properties [23,24,25]. In general, protein-based emulsifiers exhibit lower emulsifying power than small-molecule surfactants, in terms of dispersion level, but they provide emulsions with greater physical and chemical stability [26].

A main role in the chemical and physical stability of emulsified systems has been attributed to the interfacial layer, i.e., the narrow region which surrounds each emulsion droplet and is made mainly of the surfactant agents that decrease the interfacial tension and favor the fine dispersion of the two immiscible phases and its stability [26] but also of any amphiphilic molecule that is present either in the aqueous or lipidic phases and can adsorb during the emulsification process. The presence and concentration of the latter compounds at the interface have been found to affect either positively or negatively the oxidation reaction and its rate along with the physical stability of emulsions as shown in O/W emulsions made of extra-virgin olive oil or olive leaves extracts due to the presence and concentration of several bioactive compounds (e.g., oleuropein, tyrosol) for which a role as biosurface-active compounds has been found [20,23,27,28].

Despite some studies have been already carried out on the use of pistachio in formulated foods, e.g., fermented pistachio-based beverages [29,30], pistachio spread [31,32], and pistachio milk [32], no studies have reported the emulsion ability of crude pistachio oil as well as the physical and chemical stability of the oil-in-water (O/W) emulsions. Moreover, no investigations have been performed to assess the effect of emulsification on the bioactive pattern of the pistachio oil and its corresponding health properties.

Thus, this study aimed to evaluate the emulsion abilities of two commercial pistachio oils of different origins and their physical and oxidative stability upon emulsification and short-term storage (7 days, 4 °C). The fate of the main bioactive compounds in the pistachio oil due to emulsification as well as in the emulsions made thereof were also determined to evaluate the impact of the processing stresses of this technology and the effect of the emulsifier concentration.

The results could be relevant for the design and development of innovative and healthy food and non-food emulsified products.

## 2. Materials and Methods

### 2.1. Materials

Two cold-pressed pistachio oil (virgin oil) from the latest harvest of the year 2023 was obtained from different producers: Amicearth (Monghidoro, Italy) provided the oil of Spanish Kerman pistachios (PO1) and Pariani srl (Givoletto, Italy) from Italian Bronte Sicilian pistachios (“Verde di Bronte PDO, Bronte PDO Pistachio) (PO2). On arrival, oil was stored in a temperature-controlled room (15 °C) until usage. Soybean oil, used as reference, was purchased from a local store. All used reagents, sodium azide, Tween 20, phosphate buffer salts (sodium phosphate dibasic heptahydrate and sodium phosphate monobasic monohydrate), were of analytical grade and provided by Merck (Darmstadt, DE). Ultrapure water was used in all the experiments.

### 2.2. Emulsion Preparation

Oil-in-water (O/W) emulsions were prepared by homogenizing 20% (*w*/*w*) of oil pistachio PO1 or PO2, or soybean with 0.5% or 1% of Tween-20 (Tw20) as an emulsifier and the weight was continued to 100 g using sodium phosphate (0.1 M pH 7) as a buffer. A pre-emulsion was prepared using a high-speed rotor stator (Yellow Line DI 25 Basic, IKA Werke GmbH & Co, Staufen, Germany) at 13,500 rpm for 1 min. Then, the coarse emulsion was finely emulsified by a High-Pressure Homogenizer (Panda Plus 2000; GEA Niro Soavi, Parma, Italy) at a pressure of 30 MPa with a circulation time of 3 min soybean oil (SBO) was used to prepare control emulsions according to the same procedure.

### 2.3. Chemical Analysis

#### 2.3.1. Fatty Acid Profile

Fatty acid methyl esters (FAMEs) were obtained from the oil through alkaline transmethylation [33] and were analyzed by gas chromatography coupled with flame ionization detector (GC-FID) (TRACE 1300, Thermo Scientific, Waltham, MA, USA) equipped with a capillary column (Restek, Rt 2560, 100 m × 0.25 mm × 0.2 μm). The carrier gas was helium at a flow rate of 1.6 mL min^−1^. The oven temperature program was as follows: 5 min isothermal at 140 °C, then in 20 min reached the temperature of 240 °C, increasing the temperature by 4 °C every minute; finally, 15 min isothermal at 240 °C, for a total analysis of 45 min for each sample. The samples were injected into a split/splitless injection port. For identification purposes, a FAME mix standard C4–C24 Sigma Aldrich, was used. The software used for the identification and visualization of the various chromatograms was Chromeleon 7.2.10. The fatty acid composition was expressed as a percentage of fatty acid (% FA) of the total fatty acids.

#### 2.3.2. Total Phenolic Content (TPC)

Pistachio oils were subjected to a preliminary extraction following the procedure reported in [34] using 2 mL of hexane and 4 mL of a methanol/water (80:20) mixture shaken for 2 min and centrifuged. The total phenolic content (TPC) was determined on the extract using the Folin–Ciocalteu method. Briefly, 20 µL of the extracted phenols were carefully taken and placed in a new tube with 1.58 mL water and 100 µL of Folin reagent. After 7 min, 300 µL of sodium carbonate solution was added to the mixture, vortexed and left at room temperature for 30 min. The absorbance was measured at 750 nm in a VWR P4 UV-VIS spectrophotometer (Onda, UV-31 SCAN, Beijing, China). The results are expressed as mg gallic acid equivalents per g of sample, using a calibration curve of gallic acid.

#### 2.3.3. Carotenoids and Tocopherols Content

The evaluation of the carotenoids and tocopherols was carried out by an Ultra-performance liquid chromatography (UPLC) analysis by using an optimized procedure that allows their simultaneous analysis [35]. An aliquot of 60 mg of oil was inserted in 2 mL vials and subjected to extraction by 1.5 mL of acetonitrile. The extract (2 µL) was injected into UPLC equipment coupled to a photodiode array detection (PDA) and fluorimeter detector (FLR) Acquity system (Waters Corporation, Milford, CT, USA). The column was an HSS T3 C18 (100 × 2.1 mm, 1.8 µm, Waters Corporation, Milford, CT, USA). The chromatographic conditions were set accordingly as reported in Fanesi et al. [35]. PDA was set at 450 nm to detect carotenoids, while for tocopherols the FLR was at 290 nm excitation energy and 330 nm emission energy. Carotenoids and tocopherols were identified by comparison of the retention time and absorbance spectra of pure standards and quantification was performed by an external calibration curve ranging (0.5–100 µg mL^−1^) (R^2^ = 0.9977 for lutein and R^2^ = 1 for tocopherol isomers).

### 2.4. Oxidative Stability of the Oils

#### 2.4.1. Accelerated Oxidative Stability (Oxitest)

Oils were tested for oxidative stability by the Oxitest^®^ method (VELP OXITEST Reactor, VELP Scientifica Srl, Usmate (MB), Italy) according to AOCS Cd 12c-16 International Standard Procedure [36].

#### 2.4.2. Peroxide Value

Peroxide value (PV) of the pistachio oils and soybean oils was determined according to AOCS official method Cd 8b-90 and results are expressed in milli equivalent (meq) active O_2_ kg^−1^ lipids [37].

#### 2.4.3. Secondary Lipid Oxidation Products

Conjugated dienes and trienes were evaluated. A 1% (*w*/*v*) solution of the oil in iso-octane was prepared and the absorbance was measured using a spectrophotometer (VWR P4 UV-VIS, Beijing, China) using pure iso-octane as a reference. The specific extinctions at 232 nm and 268 nm are calculated for conjugated dienes and trienes and ∆K according to the official method [38].

### 2.5. Dynamic Interfacial Tension

Oil/water interfacial tension measurements were performed by Attension Sigma 700/701 tensiometer (Biolin Scientific Oy, Espoo, Finland), equipped with a Du Noüy platinum ring (diameter: 120.39 mm). Measurements were carried out on both pistachio oils (PO1 and PO2) and as reference, the soybean oil. The interfacial tension change towards the equilibrium as a function of time was determined at 20 °C. Data reported are the average of at least 3 repetitions.

### 2.6. Emulsions Characterization

#### 2.6.1. Dispersion State

Dispersion state (distribution and particle size) was used to test the emulsifying capacity of the oils and the stability of the emulsions. Particle size and distribution of the O/W emulsions was measured in water using a laser diffraction particle size analyzer (Mastersizer 3000; Malvern, Worcestershire, UK). The refractive indices of pistachio oil and soybean oil were 1.33 and 1.47, respectively. The volumetric diameter D_[4;3]_ and the specific surface area (SSA) were determined just after emulsion preparation and after 24 h storage at room temperature to evaluate the physical stability against coalescence.

#### 2.6.2. Creaming Index

After preparation, emulsions (10 mL) were added with 0.01% *w*/*w* sodium azide as an antimicrobial agent and transferred into graduate cylinders, tightly sealed, and stored at room temperature (22 ± 2 °C) for 7 days [39]. The Creaming Index (CI, %) was measured and calculated by:

CI (%) = (HL/HE) × 100,(1)

where HE is the total height of emulsions and HL is the height of the creamed layer.

### 2.7. Oxidative Status and Bioactives Content of Emulsions

The oxidative stability of the pistachio oil emulsions was determined in freshly prepared emulsions and those stored for 7 days at 4 °C by monitoring fatty acids profile and primary products of the lipid oxidation, conjugated dienes and trienes, peroxide value, total phenolic content, carotenoids and tocopherols, and color parameters.

For analysis purposes, the emulsions were preliminary subjected to two cycles of thawing and centrifuging (5000 rpm, 10 min at 20 °C) and the obtained lipids were subjected to the analyses reported in Section 2.3 and Section 2.4.

For the total phenolic content (TPC), emulsions, in order to determine the content of the phenolics of the different phases of the emulsions as well as those at the interface, were preliminarily subjected to an extraction procedure in agreement with [40]. Briefly, aliquots of 2 mL of pistachio oil emulsions were placed in a 5 mL falcon tube with 0.5 mL of acetonitrile. The sample was vigorously hand-shaken for 1 min. A 1.2 g salt mixture (MgSO_4_/NaCl 2:1, *w*/*w*) was then added and shaking was repeated under the same conditions. The resulting mixture was centrifuged at 5000 rpm for 10 min and 20 µL of supernatant was carefully taken and TPC was determined according to the Folin–Ciocalteu method as described previously.

### 2.8. Color

Color was evaluated both for the oils and the corresponding emulsions just after their preparation, using a Konica Minolta Chroma Meter CR-5 spectrophotocolorimeter (Konica Minolta, Osaka, Japan) equipped with a rectangular cell of optical glass (50 × 38; 2 mm optical path) for precise transmittance measurement of transparent liquids having high optical density. The color was determined in CIELab coordinates (L*, b* and a*) and Chroma C = (a*^2 + b*^2)^1/2 and hue angle H = arctan (b*/a*) were thereafter calculated. All determinations were performed in triplicate.

### 2.9. Statistical Analysis

All data were elaborated and analyzed using STATISTICA v. 10 (StatSoft, Inc., Tibco, CA, USA). One-way ANOVA and Tukey tests were applied to identify statistical differences (*p* < 0.05) among samples regarding chemical quality indices, fatty acids profile, color parameters, and bioactive compounds content.

## 3. Results and Discussion

### 3.1. Chemical Characterization of Pistachio Oils

Fatty acids (FAs) composition, main bioactive compounds content, and oxidative status of the two crude pistachio oils are presented in Table 1.

Some differences were found in the concentration of few fatty acids between PO1 and PO2, but overall their pattern agrees with the range of data reported in various studies and related to their origin [5,6,41,42]. In general, oleic acid is the main fatty acid, followed by the linoleic and palmitic one. Oleic acid was higher in PO2 (*p* < 0.05) than in PO1 while the latter had a significantly higher level of linoleic acid. In Bronte pistachio oil, Arena et al. [5] found 72% and 13.3%, similar to the data found in this study, while Tsantili et al. [43] found significantly lower oleic acid and higher linoleic acid contents in products of the same origin, and equal to 60.36% and 18.5% respectively. On the contrary, the authors of the latter study found a content of oleic and linoleic acid in Kerman pistachio oil of 51.60% and 27.03% [43], which aligns with our data. It has been recognized that the composition of nuts is strongly influenced by factors such as genetics, harvest season, origin, environmental conditions, soil composition, maturity level, and cultivation methods [44].

The total saturated fatty acids content is higher in PO1 than in PO2 and slightly higher than the values reported by Tsantili et al. [43], who found values of 11.40 and 11.32% in Bronte and Kerman oils with no significant differences. A lipid composition characterized by low levels of saturated fatty acids and high monounsaturated fatty acids is highly beneficial for human nutrition [45].

In Table 1 the TPC, along with the concentration of tocopherols (γ- and δ-) and lutein are reported. These bioactive compounds have been selected both to characterize the antioxidant and health potential and to trace the effect of emulsification stresses as they are the main antioxidant compounds of pistachio oils. Tocopherols and polyphenols have been recognized as the responsible compounds for the antioxidant capacity of pistachio oil [46].

The results showed no significant differences in the concentration of these bioactive phytochemicals between the two oils, being slightly higher the content of γ-tocopherol (γ-T) in PO1 and total phenolics in PO2. γ-T was reported as prominent in different varieties of virgin pistachio oil by many authors [1,14,44].

In a recent study, TPC of virgin pistachio oil was found equal to 18 mg kg^−1^ gallic acid and this value was compared to the content of virgin walnut oil (12 mg kg^−1^ gallic acid) [47]. Kornsteiner et al. [44] reported a TPC of Spanish pistachios in the range between 492–1442 mg of GAE 100 g^−1^.

Lutein was the only carotenoid detected in our study in both pistachio oils with no significant differences. Similarly, different authors have reported lutein as the main carotenoid in pistachio oil, while it was absent in the oil of other nuts such as cashews, peanuts, pecans, pines, and walnuts [1,44].

The initial oxidation status of the two pistachio oils was evaluated by determining the peroxide value and the K_232_ and K_268_ ones for the secondary oxidation products (Table 1). The peroxide value of both PO1 and PO2 was lower than 15 meqO_2_ kg^−1^ [48] falling under the upper limit value established by the Codex Alimentarius for cold-pressed oils. Moreover, for both oils K_232_ and K_268_ values were low and within the range reported in other studies [5,42,49,50].

**Figure 1:**
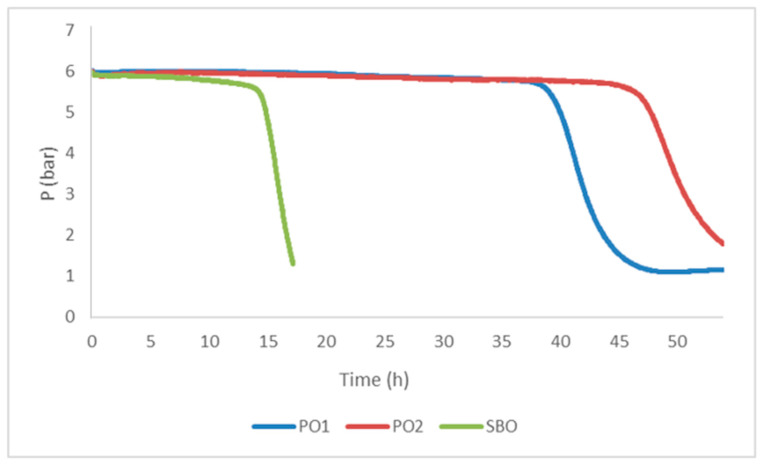
Curves obtained for the induction periods (Oxitest ^®^) of pistachio oils (PO1, PO2) compared to soybean oil (SBO).

To test and compare the oxidative stability of the pistachio oils, a validated accelerated oxidation test (Oxitest^®^) was performed to determine the induction time under high oxygen and temperature conditions; soybean oil was used as a reference (Figure 1) [51]. Overall, PO1, PO2 and SBO oils, independently on their origin and composition, showed a significantly different oxidative stability among them. Based on the induction time that corresponds to the time at which the oxygen pressure starts to decrease, both pistachio ones (46 h and 39 h for PO2 and PO1, respectively) showed a significantly higher oxidative resistance than SBO (Figure 1).

Moreover, the steep decrease of the oxygen pressure of the SBO sample after the induction period corresponds to a rapid oxidative degradation which results different from the trend observed in both pistachio oils that, on the contrary, showed a slower decrease to indicate a higher resistance to oxidation. This result could be attributed to the lower content of PUFAs in PO2, as it was found to have similar levels of antioxidants, including TPC, lutein, and tocopherols.

The presence and concentration of other phytochemicals with antioxidant ability (e.g., sterols, chlorophylls, other carotenoids) and/or the specific phenolic pattern not determined in this study, could be also considered as additional factors determining a higher oxidation resistance. Similar induction time data for virgin pistachio oil were reported by other authors. Fregapane et al. [47] found an induction time of 40 h when the pistachio oil was heated to 100 °C to determine oxidation by Rancimat. In another study, the oxidation induction period of a 3.5 g pistachio oil sample stored at 20 °C was longer than 30 h at 100 °C under an airflow of 10 L h^−1^ [6]. The oxidative stability of cold-pressed oils has been related to the fatty acid content and endogenous antioxidants such as tocopherols [52]; pistachio oil has been reported as one of the most stable nut oils [6,46].

### 3.2. Dynamic Interfacial Tension

**Figure 2:**
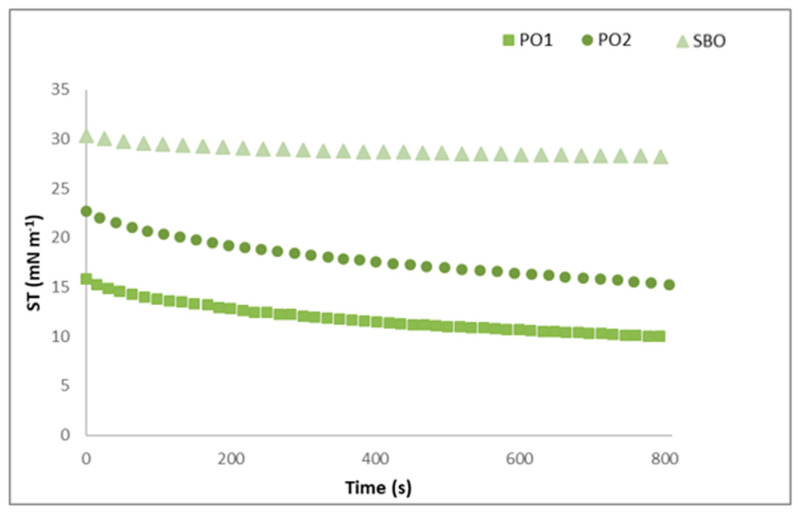
Dynamic interfacial tension (ST: surface tension) of pistachio (PO1, PO2) oils and soybean oil (SBO, reference) against water, as a function of time.

In Figure 2 the dynamic interfacial tension, determined using the Du–Noüy ring method, of the two pistachio oils against water as a function of time is shown and trends are compared with that of the commercial soybean oil. The dynamic interfacial tension of oils is an important index of their performances during emulsification and emulsion stability, both from a physical and chemical perspective [53].

The two pistachio oils showed a similar trend in terms of time-dependence decrease, which was more relevant at a short time of observation followed by a slower reduction towards an asymptotic response over time. On the contrary, soybean oil showed a limited reduction at shortest time of observation reaching a pseudo-equilibrium quite soon. Overall, the three oils show significant differences in their interfacial tension values of several mN m^−1^ that, at equilibrium were equal to 10.7 (±0.4), 14.07 (±1.0) and 28.2 (±0.62) mN m^−1^. Moreover, different diffusion kinetics of the bio-surfactants at the oil/water interface were observed, index of a different composition of the oils.

The results highlight how the interfacial tensions of pistachio oils can vary depending on their composition, which is related to several parameters, like origin, cultivar as well as agronomic and processing variables. Similarly, as previously reported, extra-virgin olive oil samples also exhibit differences in interfacial tension of several mN m^−1^, reflecting variations in their composition [53]. 

The interfacial properties of dietary oils do not depend on their triacylglycerols composition that, in general, leads to similar interfacial tension values, but they reflect the presence and concentration of minor surface-active components able to adsorb at the interface [54]. Several studies carried out on crude vegetable oils, as well as physically or chemically refined lipids, have demonstrated that phenolic compounds are the minor components with the greatest influence on interfacial tension against water, followed by free fatty acid, linoleic acid in triglyceride and phospholipids [55]. The lower interfacial tension of PO1 compared to PO2 could be related to the higher concentration of PUFAs (Table 1) along with the presence and/or content of other specific amphiphilic phenolic compounds despite that were not determined in this work. It is known that gallic acid, catechin and quercetin are directly involved in the decrease of the interfacial tension at the oil-water interface [20,55]. Although commercial soybean oil contains a high content of PUFAs (generally > 50%), several studies have shown that it has a very low total phenolic content, ranging from 3 to 4 mg GAE g^−1^ of oil [55,56].

### 3.3. Emulsion Physical and Chemical Properties and Stability

Emulsions based on PO1 and PO2 were formulated and stabilized by Tw20, used at two concentrations (0.5 and 1% *w*/*w*); the droplet size (D_[4;3]_) of the emulsified samples after preparation and after 24 h, together with the specific surface area (SSA) and creaming indices (CI) are reported in Table 2. Homogenization conditions and emulsifier concentration being equal, the use of PO1 and PO2 oils unexpectedly caused the formation of larger droplet sizes when compared to SBO, that was reflected also in SSA results. Thus, despite the lower interfacial tension values of the two pistachio oils with respect to SBO, under dynamic conditions and in presence of minor surface-active compounds, PO1 and PO2 showed a reduced emulsifying capacity likely due to competitive phenomena at the oil/water interface between the amphiphilic compounds present in the pistachio oils and the Tw20 [57]. PO1 was found to exert a better emulsifying capacity when compared to PO2, irrespective of Tw20 concentration.

After 24 h, the emulsions formulated with PO showed a decrease in the D_[4;3]_ values, which was likely ascribable to flocculation phenomena in the systems freshly prepared that reversed during the 24 h storage time, whilst the SBO-based emulsions showed a significant increase in droplet size after 24 h, suggesting the occurrence of aggregation mechanisms among the droplets.

Long term stability is a prerequisite condition for many emulsions; therefore, the creaming index (CI), a parameter commonly used to monitor the physical stability of multiphasic systems, was monitored over a storage of 7 days. Indeed, creaming is considered as a precursor of coalescence which can ultimately lead to phase separation; moreover, the creaming index can also give useful indications about droplets agglomeration in an emulsion, the higher the agglomeration the higher would be the creaming index [58]. According to CI results, emulsions with PO1 and PO2 stabilized by Tw20 (0.5%) demonstrated higher values and, thus, lower resistance to creaming compared to emulsions prepared with SBO, while no main differences were seen when Tw20 was present in the lowest amount.

**Figure 3:**
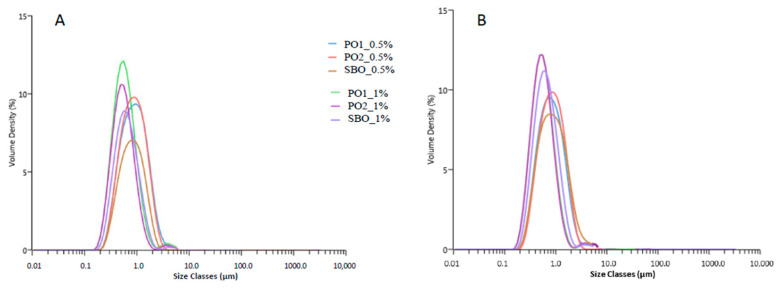
Particle size distributions of pistachio oil (PO1, PO2) and of soybean oil (SBO) emulsions, just after preparation (**A**) and after 24 h (**B**).

The droplet size distribution of the freshly prepared emulsions (A) and after 24 h (B) are shown in Figure 3. Irrespective of the type of oil, a higher concentration of Tw20 led to finer and uniformly dispersed emulsions as could be noticed by their narrower droplet size distribution despite the presence of a small population fraction of particles with larger sizes is also present. On the contrary, at the lowest Tw20 concentration, wider droplet size distributions were obtained with no tails on larger sizes. The presence of larger particles usually represents a criticism as they can promote particles agglomeration and merging phenomena at the expense of smaller particles; this may explain why in the PO1 and PO2 emulsions formulated with Tw20 (1%) higher CI values were found. The distribution of fat droplets in both pistachio oil emulsions was stable after 24 h presenting the same trend as in SBO emulsions. Similarly, mayonnaise-like emulsions made from purified olive oil were characterized also by a monomodal droplet distribution and a droplet size with average values ranging between 3.30 and 3.50 μm [23]. In contrast, the distribution profile of droplet size for Brazil nut oil emulsions presented bimodal behavior [59].

In Table 3, the fatty acid composition of the emulsions prepared with two different pistachio oils (PO1 and PO2) at two different concentrations of Tw20 (0.5% and 1%) on day 1 and day 7 stored at 4 °C, is reported. The fatty acid composition remained stable in all prepared emulsions compared to that of their corresponding initial oils. In terms of oxidation, PO2 emulsions were found to be higher in PV values compared to PO1, in agreement with the PV values determined in the corresponding crude oils and data were not affected by Tw20 concentration. These results confirm the pro-oxidant effect of homogenization which can induce the oxidation of unsaturated fatty as previously reported in literature [60,61]. However, conjugated trienes (i.e., K_268_ values) remained stable after emulsification, indicating that secondary oxidation products are not significantly formed.

**Figure 4:**
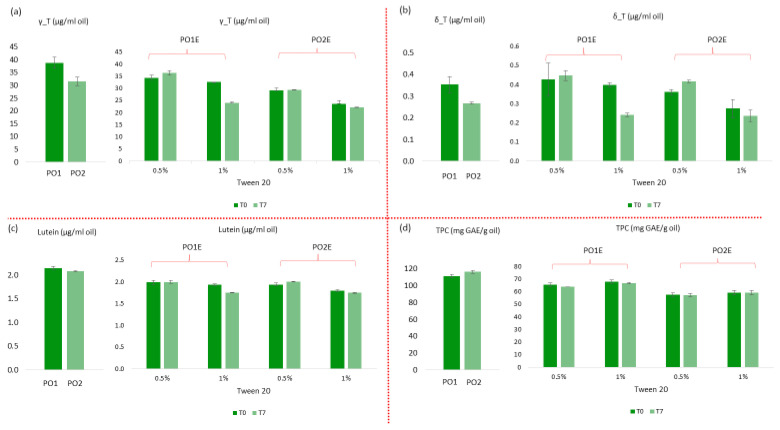
Content of (**a**) γ-T (γ-Tocopherol); (**b**) δ-T (δ-Tocopherol); (**c**) lutein; (**d**) TPC (total phenol content) in the crude oils (PO1 and PO2) and in the dispersed phase of PO1E and PO2E emulsions made of 0.5 and 1% Tw20 over storage.

The effect of emulsification and emulsifier amount on the content of bioactive compounds and on their chemical stability over a storage of 7 days at 4 °C was also studied and results are reported in Figure 4(a–d); data are shown and compared with respect to their amount in the crude oil. It is evident that the emulsification process had a significant impact on the TPC values which resulted in significantly lower in both the set of emulsions when compared with the crude oil, likely due to a partitioning effect of the amphiphilic and polar phenolic compounds into the aqueous phase [20]. Pistachio oils were indeed shown to be rich in phenolic acids, like gallic and caffeic acids, and flavonoids glucosides, that can be considered polar amphiphilic structures with a high affinity towards water [62]. Over storage, the total content of phenolic compounds remained stable, despite the pro-oxidant activity of emulsification, widely reported in literature [60,61] and evidenced by the increase in PV in both the oils with respect to the crude oils. The hydrophobic compounds γ-T, lutein and δ-T were not significantly influenced neither by emulsification nor by emulsifier concentration but they were negatively affected by storage, especially in PO1 stabilized by Tw20 1% (*w*/*w*).

### 3.4. Color Properties

Color properties of pistachio oils and the corresponding emulsions, just after preparation, are summarized in Table 4. It has been reported that the emerald-green peculiar colour of pistachio seeds is due to the presence and concentration of chlorophyll a, chlorophyll b, β carotene and lutein [10,63]. The negative value of a* (−5.79) associated to a rather high b* value of PO1 indicates a yellow colour with a significant greenish tonality related to the presence of chlorophylls present in the oil. The literature reports colour data for pistachio oils from 20 different cultivars indicating negative a* values ranging from −7.56 to −8.31 [64]. Higher a* values, near to zero, were observed in PO2, not significantly affecting both the corresponding chroma and hue values, result that could be related to different causes.

Chlorophylls present in pistachio are highly sensitive to technological stresses and oxidation and converted into derivatives, like pheophytins and phyropheophytins [17]. Less green pigments could be related to a different origin, pigment profile, or ripening level of the seeds corresponding to a higher level of their degradation [10].

When chromophore substances are present in the dispersed phase, a fraction of the light wave is absorbed. The extent of absorption depends on the concentration and absorptivity of the chromophores, such as chlorophylls and carotenoids, as well as the wavelength of the light used [65]. PO1 and PO2 emulsions are characterized by a lower lightness and chroma (C) values compared to their corresponding oils with no main differences between the L* values of the former ones. In general, in dispersed O/W systems, smaller droplet sizes may amplify the light scattering, increasing brightness while diminishing chromaticity, thereby a direct impact on visual properties of emulsions could be observed [65]. On the other hand, it has to be also considered that opacity and the decrease of color saturation has been recognised as one of the main characteristics of the emulsions as determined by the particle concentration, size, refractive index contrast, and presence of any chromophores that absorb light [66]. The lower brightness (higher L*) and reduced b* values observed in the emulsions compared to the corresponding oils could be attributed to the combined effects of light scattering and absorption. However, the two series of pistachio oil emulsions could be considered similar in terms of their dispersion degree, which is one of the key factors affecting lightness in O/W emulsions, other parameters being equal [58]. Emulsification determined a significant increase of a* and decrease of b* in both series of emulsions in respect to the corresponding crude oils with no effect due to the Tw20 concentration used. The hue decrease is higher in the PO2 series than the PO1. Some studies evidenced the chlorophyll degradation into yellow-brown derivatives due to process stresses chlorophyll [17,67] the formation of lipid oxidation products can also cause yellowing or browning [68]. On the other hand, pigments partitioning among the three phases of the O/W emulsions (aqueous, oil, interface) during emulsification could have also an effect on the final colour properties of the emulsions and this aspect needs additional investigation.

## 4. Conclusions

This study highlights the potential of pistachio oil as a lipid phase in the development of food emulsions. The unique and complex chemical properties resulted not impairing the formation of fine and stable emulsions. PO1 and its corresponding emulsions demonstrated higher levels of bioactive compounds compared to PO2. While the origin of the pistachio oil and its composition did not significantly affect the emulsifying properties, further studies are needed to explore the role of specific naturally present phytochemicals in influencing the emulsifying capacities and both physical and chemical stability of the corresponding emulsions.

This study also revealed the negative impact of emulsification on the oil’s oxidation status by the decrease of TPC and the increase of the peroxide value of the dispersed oil phase compared to the crude oil. The effect of emulsification process conditions on the physical, chemical, and sensory properties of the O/W pistachio oils will be part of future studies. Understanding the impact of phenolic compound partitioning into the aqueous phase on bioactivity and health claims requires further investigation also in the perspective of a longer stability of pistachio oil emulsions to be exploited for commercial food and non-food applications with expected long-term shelf-life. 

Overall, these findings confirm the feasibility of using pistachio oil to develop O/W emulsions rich in bioactive compounds, paving the way for new opportunities in creating healthy emulsified foods and ingredients.

## Figures and Tables

**Table 1:** Fatty acid (FA) composition (% wt of total FAs), total phenolic content (TPC), carotenoids and tocopherols content, and oxidative status indices. Results are reported as means ± standard deviation (*n* = 3). Cm:n Dx; m = number of carbon atoms, n = number of double bonds, x = position of double bonds.

Parameter	PO1	PO2
C14:0	0.08 ± 0.01 ^a^	0.13 ± 0.01 ^a^
C16:0	11.09 ± 0.13 ^a^	9.40 ± 0.07 ^b^
C16:1	0.98 ± 0.02 ^a^	0.37 ± 0.31 ^a^
C17:1	0.20 ± 0.01 ^a^	0.10 ± 0.01 ^a^
C18:0	0.84 ± 0.54 ^a^	2.09 ± 0.01 ^b^
C18:1	57.18 ± 0.11 ^a^	72.94 ± 0.26 ^b^
C18:2 [ω6]	29.23 ± 0.19 ^a^	14.32 ± 0.19 ^b^
C18:3 [ω3]	0.29 ± 0.02 ^a^	0.45 ± 0.01 ^a^
C20:0	0.10 ± 0.01 ^a^	0.21 ± 0.05 ^a^
Σ SFA	12.12 ± 0.3 ^a^	11.82 ± 0.2 ^a^
Σ MUFA	58.36 ± 0.1 ^a^	73.41 ± 0.3 ^b^
Σ PUFA	29.53 ± 0.1 ^a^	14.76 ± 0.1 ^b^
TPC (mg GA/g oil)	110.96 ± 1.91 ^a^	115.96 ± 1.91 ^a^
γ-T (μg/mL)	38.83 ± 2.15 ^a^	31.58 ± 1.72 ^a^
δ-T (μg/mL)	0.35 ± 0.04 ^a^	0.27 ± 0.01 ^a^
Lutein (μg/mL)	2.11 ± 0.1 ^a^	2.07 ± 0.02 ^a^
PV (meqO_2_/kg)	1.95 ± 0.07 ^a^	6.97 ± 0.02 ^b^
K_232_	2.15 ± 0.03 ^a^	2.45 ± 0.03 ^a^
K_268_	0.21 ± 0.01 ^a^	0.19 ± 0.01 ^a^
∆K	0.004 ± 0.01 ^a^	0.005 ± 0.01 ^a^

(γ-T: γ-tocopherol; δ-T: δ-tocopherol; PV: peroxide value; K_232_: conjugated dienes; K_268_: conjugated trienes); ^a,b^: Mean values in each row followed by different superscript letters are significantly different for *p* < 0.05.

**Table 2:** Dispersion degree of o/w emulsions prepared with pistachio oils (PO1, PO2) and soybean oil (SBO) at t = 0 and t = 24 h and creaming index (t = 7 days).

Parameter	Time	SBO		PO1		PO2	
		Tw_20_—0.5%	Tw_20_—1%	Tw_20_—0.5%	Tw_20_—1%	Tw_20_—0.5%	Tw_20_—1%
D_[4;3]_ (μm)	T_0_	1.2 ± 0.12 ^b^	0.6 ± 0.02 ^c^	1.5 ± 0.08 ^b^	1.1 ± 0.05 ^b^	2.5 ± 3.2 ^a^	2.1 ± 0.1 ^ab^
	T_24_	2.0 ± 0.17 ^a^	1.5 ± 0.22 ^b^	1.8 ± 0.36 ^a^	0.7 ± 0.13 ^c^	1.4 ± 0.07 ^b^	1.3 ± 0.1 ^b^
SSA (m^2^ g^−1^)	T_0_	9867 ± 8.9 ^b^	14,218 ± 19.2 ^a^	8460 ± 19.7 ^c^	12,432 ± 4.5 ^ab^	6344 ± 17.8 ^d^	12,932 ± 15.7 ^ab^
	T_24_	8678 ± 31.4 ^c^	11,100 ± 68.2 ^ab^	9109 ± 51.4 ^b^	13,110 ± 4.5 ^a^	8841 ± 14.4 ^c^	12,964 ± 28.8 ^ab^
Creaming index (%)		8.2 ± 0.87 ^b^	3.8 ± 0.58 ^c^	11.4 ± 0.23 ^a^	3.8 ± 0.02 ^c^	10.1 ± 0.01 ^a^	4.4 ± 0.89 ^c^

^a–c^: Mean values of data at T_0_ and T_24_ in each row followed by different superscript letters present significant differences for *p* < 0.05.

**Table 3:** Fatty acids and oxidative status indices of o/w emulsions prepared with pistachio oils (PO1, PO2) at day 1 (D1) and after 7 days (D7) of storage.

Parameter	Day	PO1 Emulsions		PO2 Emulsions	
		Tw_20_—0.5%	Tw_20_—1%	Tw_20_—0.5%	Tw_20_—1%
Σ SFA	D1	12.8 ± 0.01 ^a^	12.7 ± 0.01 ^a^	12.2 ± 0.02 ^a^	11.6 ± 0.01 ^a^
	D7	12.6 ± 0.01 ^a^	12.3 ± 0.01 ^a^	11.5 ± 0.01 ^a^	11.5 ± 0.01 ^a^
Σ MUFA	D1	58 ± 0.01 ^a^	58.2 ± 0.02 ^a^	72.6 ± 0.01 ^b^	73.8 ± 0.01 ^b^
	D7	58.2 ± 0.01 ^a^	58.3 ± 0.02 ^a^	73.6 ± 0.02 ^b^	73.6 ± 0.01 ^b^
Σ PUFA	D1	29.1 ± 0.01 ^a^	29.1 ± 0.02 ^a^	15.2 ± 0.01 ^b^	14.6 ± 0.03 ^b^
	D7	29.2 ± 0.01 ^a^	29.4 ± 0.02 ^a^	14.8 ± 0.04 ^b^	14.9 ± 0.01 ^b^
PV (meqO_2_ kg^−1^)	D1	5.46 ± 1.1 ^a^	5.11 ± 1.1 ^a^	11.30 ± 0.9 ^b^	11.79 ± 1.3 ^b^
	D7	5.99 ± 1.5 ^a^	5.95 ± 1.0 ^a^	11.84 ± 1.1 ^b^	11.43 ± 1.5 ^b^
K_232_	D1	2.62 ± 0.01 ^a^	2.46 ± 0.04 ^a^	2.72 ± 0.01 ^a^	2.53 ± 0.02 ^a^
	D7	2.43 ± 0.21 ^a^	2.33 ± 0.15 ^a^	2.83 ± 0.15 ^a^	2.72 ± 0.11 ^a^
K_268_	D1	0.21 ± 0.02 ^a^	0.19 ± 0.02 ^a^	0.20 ± 0.04 ^a^	0.19 ± 0.01 ^a^
	D7	0.19 ± 0.30 ^a^	0.17 ± 0.28 ^a^	0.20 ± 0.25 ^a^	0.20 ± 0.25 ^a^
∆K	D1	0.003 ± 0.1 ^a^	0.002 ± 0.02 ^a^	0.005 ± 0.01 ^a^	0.003 ± 0.02 ^a^
	D7	0.004 ± 0.01 ^a^	0.002 ± 0.02 ^a^	0.003 ± 0.2 ^a^	0.001 ± 0.2 ^a^

(PV: peroxide value; K_232_: conjugated dienes; K_268_: conjugated trienes); ^a,b^: Mean values of data in rows followed by different superscript letters are significantly different for *p* < 0.05.

**Table 4:** Colorimetric parameters of PO1 and PO2 pistachio oils and their corresponding PO1E and PO2E emulsions.

Sample	Tw20	L*	a*	b*	C	Hue
PO1		83.84 ± 1.56 ^a^	−5.79 ± 2.68 ^c^	116.04 ± 1.63 ^a^	116.21 ± 1.73 ^a^	92.85 ± 1.29 ^a^
PO1E	0.5%	20.27 ± 0.01 ^b^	0.08 ± 0.2 ^b^	33.93 ± 0.01 ^b^	33.93 ± 0.01 ^b^	89.87 ± 0.01 ^b^
	1%	20.01 ± 0.01 ^b^	0.72 ± 0.01 ^b^	33.61 ± 0.01 ^b^	33.62 ± 0.01 ^b^	88.78 ± 0.01 ^b^
PO2		80.41 ± 0.33 ^a^	0.98 ± 0.12 ^b^	111.91 ± 8.50 ^a^	110.06 ± 7.08 ^a^	89.5 ± 0.07 ^b^
PO2E	0.5%	19.14 ± 0.06 ^b^	6.33 ± 0.01 ^a^	32.19 ± 0.01 ^b^	32.81 ± 0.01 ^bc^	78.88 ± 0.01 ^c^
	1%	18.05 ± 0.01 ^bc^	7.08 ± 0.01 ^a^	30.44 ± 0.01 ^bc^	31.25 ± 0.01 ^c^	76.91 ± 0.01 ^c^

^a–c^: Mean values in each column followed by different superscript letters present significant differences for *p* < 0.05.

## Data Availability

The original contributions presented in this study are included in the article. Further inquiries can be directed to the corresponding authors.
